# EMMPRIN promotes spheroid organization and metastatic formation: comparison between monolayers and spheroids of CT26 colon carcinoma cells

**DOI:** 10.3389/fimmu.2024.1374088

**Published:** 2024-04-25

**Authors:** Gabriele Feigelman, Elina Simanovich, Phillipp Brockmeyer, Michal A. Rahat

**Affiliations:** ^1^ Immunotherapy Laboratory, Research Laboratories, Carmel Medical Center, Haifa, Israel; ^2^ Department of Immunology, Rappaport Faculty of Medicine, Technion-Israel Institute of Technology, Haifa, Israel; ^3^ Department of Oral and Maxillofacial Surgery, University Medical Center Göttingen, Göttingen, Germany

**Keywords:** spheroids, CT26 cells, EMMPRIN/CD147, dormancy, EMT, angiogenesis, proliferation

## Abstract

**Background:**

*In vitro* studies often use two-dimensional (2D) monolayers, but 3D cell organization, such as in spheroids, better mimics the complexity of solid tumors. To metastasize, cancer cells undergo the process of epithelial-to-mesenchymal transition (EMT) to become more invasive and pro-angiogenic, with expression of both epithelial and mesenchymal markers.

**Aims:**

We asked whether EMMPRIN/CD147 contributes to the formation of the 3D spheroid structure, and whether spheroids, which are often used to study proliferation and drug resistance, could better model the EMT process and the metastatic properties of cells, and improve our understanding of the role of EMMPRIN in them.

**Methods:**

We used the parental mouse CT26 colon carcinoma (CT26-WT) cells, and infected them with a lentivirus vector to knock down EMMPRIN expression (CT26-KD cells), or with an empty lentivirus vector (CT26-NC) that served as a negative control. In some cases, we repeated the experiments with the 4T1 or LLC cell lines. We compared the magnitude of change between CT26-KD and CT26-WT/NC cells in different metastatic properties in cells seeded as monolayers or as spheroids formed by the scaffold-free liquid overlay method.

**Results:**

We show that reduced EMMPRIN expression changed the morphology of cells and their spatial organization in both 2D and 3D models. The 3D models more clearly demonstrated how reduced EMMPRIN expression inhibited proliferation and the angiogenic potential, while it enhanced drug resistance, invasiveness, and EMT status, and moreover it enhanced cell dormancy and prevented CT26-KD cells from forming metastatic-like lesions when seeded on basement membrane extract (BME). Most interestingly, this approach enabled us to identify that EMMPRIN and miR-146a-5p form a negative feedback loop, thus identifying a key mechanism for EMMPRIN activities. These results underline EMMPRIN role as a gatekeeper that prevents dormancy, and suggest that EMMPRIN links EMT characteristics to the process of spheroid formation.

**Conclusions:**

Thus, 3D models can help identify mechanisms by which EMMPRIN facilitates tumor and metastasis progression, which might render EMMPRIN as a promising target for anti-metastatic tumor therapy.

## 1 Introduction

Colorectal cancer (CRC) is a prevalent type of cancer, and CRC metastatic lesions that frequently spread to the liver and lung, are considered the second leading cause of mortality (1). Metastasis is already present in 20% of the patients when they are first diagnosed, and 60% of the patients without metastasis develop it within five years (2). In some cases, primary tumors or metastases can be removed by surgery, whereas in non-operable tumors or metastatic lesions, chemotherapy with or without VEGF inhibitors is employed (3). However, these approaches fail when the metastatic cells develop drug resistance (4). Moreover, some treatment modalities may promote metastatic cells to exit their dormant state and generate active lesions in remote organs (5). Thus, better understanding of the mechanisms promoting the metastatic cascade is needed.

Epithelial cells are tightly attached to their neighboring cells. To start invasion and the metastatic cascade, they must first detach and enhance their motility by activating the epithelial-to-mesenchymal transition (EMT) program. The disseminating cells lose their adhesion to other cells, intravasate into lymph or blood vessel, circulate and then extravasate. The small percentage of cells that survive this journey, lodge themselves in the distant organ, where they can lie dormant for months or years (6, 7). To escape dormancy, the cells must undergo the reverse mesenchymal-to-epithelial transition (MET) process, that allows their proliferation and the generation of macro-metastases (8). Hence, metastatic cells are plastic and at different stages of the process epithelial and mesenchymal markers can be expressed at different degrees (hybrid EMT) (9, 10). E-cadherin is considered an epithelial marker, and its loss of expression together with enhanced expression of vimentin and enhanced expression of the EMT transcription factors (EMT-TFs) Snail/SNAI1, Slug/SNAI2, Zeb1 or Twist1 mark a more mesenchymal phenotype (11). Dormancy is also associated with reduced proliferation and angiogenesis and increased resistance to chemotherapeutic drugs (9, 12). The dormancy marker orphan nuclear receptor N2RF1, which is highly expressed in such cells, also activates the cyclin-dependent kinases p21 and p27, as well as genes related to stemness and cell survival, such as SOX2, NANOG (13–15).

Most *in vitro* studies still use two-dimensional (2D) monolayers to evaluate tumor cell characteristics, functions, and behavior. However, these conditions do not accurately simulate the complexity, the heterogeneity, and the tumor microenvironment (TME) of solid tumors. Three-dimensional (3D) structures, such as spheroids, are closer to solid tumor in their organization and cell-cell interactions. The spherical structure creates gradients of oxygen and nutrients that generate heterogeneity in the rate of proliferation and metabolism of the spheroid cells, and in their expression of genes (16, 17). Although these 3D structures are only an intermediate model of the tissue and do not fully model the intricate interactions between cells and their neighboring cells or the tissue-specific extracellular matrix (ECM) components (18), spheroids have become a relevant *in vitro* model to study the development of resistance to chemotherapeutic treatment (19) and a high throughput screening platform for drugs (20). However, other than E-cadherin and integrins, the proteins mediating the cell-cell interactions that form the spheroid structure are still not well characterized (21).

Many studies have revealed that spheroids can acquire EMT phenotypes that allow them to be more aggressive, and exhibit enhanced drug resistance, increased migration and invasion, and increased expression of mesenchymal proteins, such as vimentin, compared to their 2D monolayer counterparts (22–24). Moreover, the EMT program is critical to the formation of spheroids, as demonstrated in ascites-derived cells from ovarian cancer patients (25). However, proteins that mediate the formation of spheroids and form the link to EMT are still not fully identified.

EMMPRIN/CD147 is a multifunctional glycoprotein that is overexpressed in many types of cancers and participates in multiple cellular processes, due to its interactions with a variety of proteins (26). It is known as a pro-angiogenic protein that can induce both VEGF and matrix metalloproteinases (MMPs) (27, 28), as well as an adhesion molecule that can prevent anoikis (29, 30). Moreover, EMMPRIN functions as a hub protein that stabilizes large protein complexes (e.g., CD44, MCT1/4, CD99, integrins, P-gp), and is therefore implicated as a regulator of tumor cell proliferation, metabolism, lactate efflux, metastasis and drug resistance (31, 32). EMMPRIN is also associated with the EMT process and with stemness (33), as it is regulated by Slug (34), and can enhance β-catenin phosphorylation and suppress E-cadherin membranal expression (35). However, we are not aware of any study that directly linked EMMPRIN to the ability to form spheroids.

In this study, we asked whether EMMPRIN participates in the formation of spheroid structures, and whether its expression affects the EMT process and the metastatic outbreak. To this end, using the parental mouse colon carcinoma cell line CT26 (CT26-WT) and its EMMPRIN knocked-down counterpart (CT26-KD), we demonstrate that reduced EMMPRIN expression changed spheroid morphology, inhibited proliferation and angiogenesis, and enhanced drug resistance, invasiveness, the EMT status, and dormancy. All these effects could be better seen in spheroids rather than in monolayers. The reduced EMMPRIN expression also inhibited the ability of cells to form metastatic-like lesions in an *in vitro* 3D metastatic outbreak assay. We also demonstrate that EMMPRIN and miR-146a-5p form a negative feedback loop, thus identifying a key mechanism for EMMPRIN activities.

## 2 Materials and methods

### 2.1 Preparation of the EMMPRIN knocked-down cells

**Figure 1:**
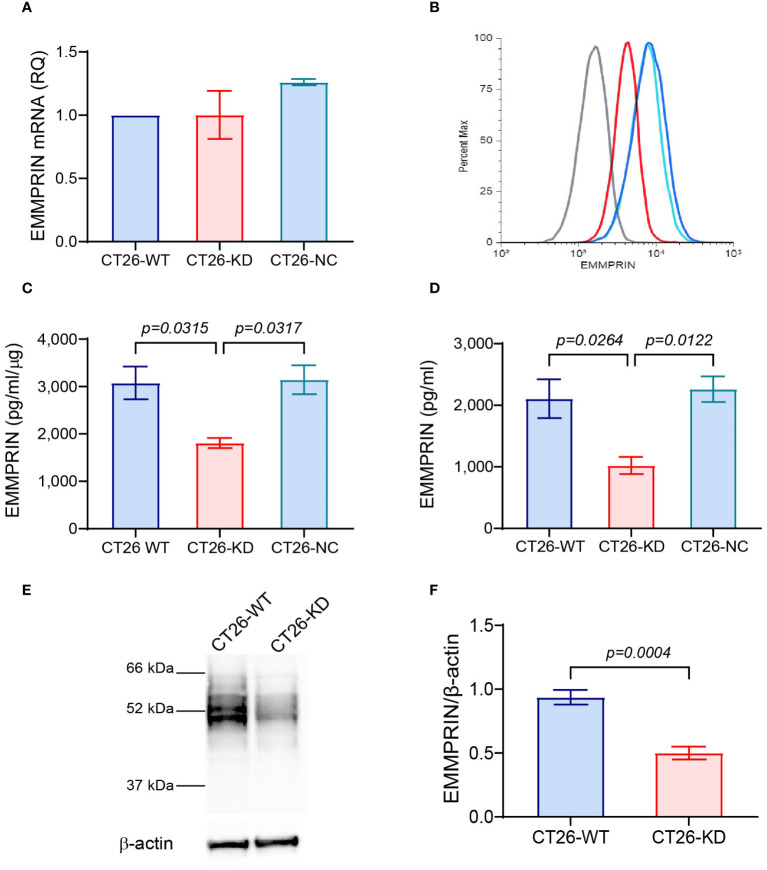
Validation of EMMPRIN expression in CT26-KD cells. The parental CT26 cells (CT26-WT) were infected with the lentivirus vector carrying EMMPRIN siRNA sequences (CT26-KD), or with the empty vector as negative control (CT26-NC). All three cells were each plated in 24-well plates (8×10^4^ cells/well/300μL) in full medium for 48 h. Total RNA or protein were extracted from the cells and supernatants were collected. **(A)** EMMPRIN mRNA was amplified and determined by qPCR (n=4). **(B)** Expression of membranal EMMPRIN was determined by flow cytometry (n=3). Grey line, isotype control; blue line, CT26-WT; light blue, CT26-NC; red line, CT26-KD cells. **(C)**. EMMPRIN protein expression in cell lysates (n=4), and **(D)** Secreted EMMPRIN levels in supernatants were evaluated by ELISA (n=9). Data are presented as means ± SEM and are analyzed using one-way ANOVA followed by the Bonferroni’s *post hoc* test. **(E)** Representative western blot analysis of EMMPRIN expression in CT26-WT and CT26-KD cells, and **(F)** its quantitation (n=5). Data are presented as means ± SEM, and analyzed using the unpaired two-tailed student *t* test.

To knock-down EMMPRIN expression, we used the procedure described before (36). Briefly, the lentivirus vector with four mouse EMMPRIN siRNA sequences (CT26-KD) or the empty vector (CT26-NC, both from Applied Biological Materials, Richmond, BC, Canada) were used to infect the parental CT26 (CT26-WT) cell line at a multiplicity of infection (MOI) 135 with polybrene (8 μg/ml, Merck, Rathway, NJ, USA). The four siRNA sequences are given in **Supplementary Table S1**. After infection, cells were grown for a total of 3 weeks in full medium with puromycin (10 μg/ml) to select for positive cells, and the medium was replaced every 3-4 days. Because the vector did not contain the GFP marker that is known to be immunogenic (37), we used the limiting dilution approach to isolate sub-clones that were similar in respect to EMMPRIN expression. These selected sub-clones were characterized for their EMMPRIN expression at the mRNA levels, membranal and secreted protein expression (**Figure 1**). The 4T1-KD and LLC-KD cells were generated in the same manner.

### 2.2 Cell cultures and preparation of 2D monolayers and 3D monoculture spheroids

The murine colon carcinoma cell line CT26 (ATCC CRL-2638) was cultured in RPMI-1640 medium with 10% fetal calf serum (FCS), 1% penicillin/streptomycin, 1% non-essential amino acids, 1% amphotericin B, 1% pyruvate and 1% L-Glutamine (full medium, all reagents from Biological industries, Beit Ha’emek, Israel). The mouse brain endothelial cell line bEND3 (ATCC CRL-2299) was cultured in high glucose DMEM, with 10% FCS, 1% penicillin/streptomycin, and 1% glutamine. The murine mammary carcinoma 4T1 (ATCC CRL-2539) and the Lewis lung carcinoma (LLC, ATCC CRL-1642) cell lines were grown in Dulbecco’s modified Eagle’s medium (DMEM) with 10% FCS, 1% penicillin/streptomycin, 1% amphotericin B, and 1% L-Glutamine. All cells were split every 3–4 days at a ratio of 1:4 using trypsin-EDTA, and were used at passages 3–15. All cells were routinely checked for the presence of mycoplasma.

To form a monolayer, CT26-WT, CT26-KD or CT26-NC cells (8x10^4^ cells/well) were incubated overnight in 300µl full medium in a 24 well plate to allow their adherence. Then the medium was replaced with 300µl serum starvation medium to avoid the masking of signals, and the cells were incubated for 48 hours. To form 3D monoculture spheroids, we used the scaffold-free liquid overlay method, where CT26-WT, CT26-KD or CT26-NC cells (5x10^3^ cells/100 μl) were seeded in full medium in a U-shaped 96-well plate that was previously coated with 1% agarose (50 μl/well in PBS). Cells were incubated for 72 hours in full medium, to allow them to mature and form tight spheroids.

### 2.3 Cell viability assay and cell proliferation

To assess viability, CT26-WT, CT26-KD and CT26-NC cells were seeded as monolayers in 96-well plates (10^4^ cells/well/100μl of serum starvation medium) for 48 hrs. Viability of the cells was assessed by adding 10µL of the cell counting kit 8 reagent (CCK-8, Abcam, Cambridge, United Kingdom) and incubating for additional 2 hrs. Alternatively, cells were seeded as spheroids (5x10^3^ cells/100μl full medium) and incubated for 72 hrs. Then the spheroids in 100μl medium were moved to another well and 10μl CCK-8 were added. Cell viability was determined according to the absorbance measured at 450 nm with 620 nm reference, resulting from the reduction of the tetrazolium salt WST-8 in the mitochondria of viable cells, and normalized to the CT26-WT cells.

Additionally, to measure cell proliferation we used the BrdU Cell Proliferation Assay kit (Biovision/Abcam, Waltham, MA) according to the manufacturer’s instructions. This kit is based on the detection of the DNA-incorporated pyrimidine analogue using a primary mouse anti-BrdU antibody and an anti-mouse HRP-linked secondary antibody. The color developed by the HRP substrate and read at 450nm is proportional to the BrdU incorporated and to number of proliferating cells.

### 2.4 Cell morphology

To observe changes in morphology, cells (3x10^3^ cells/well) were seeded in a 96-well plate in 100μL of full medium as monolayers. After 48 h of incubation, cells were fixed with 50μL of cold methanol for 5 min, dried in air for 5 min, and washed three times with PBS. Then cells were stained with 50μL of 0.1% of crystal violet solution (Merck) and incubated for 20 min. in room temperature. Excess of the crystal violet stain was removed with three washes with double distilled water (DDW). Morphological changes were investigated under light microscopy, and the aspect ratio (the ratio between the length and the width of the cells) indicating the extent of cell elongation was determined using the ImagePro Plus 4.5 software (Media Cybernetics, Inc., Rockville, MD, USA). The morphology of cells seeded in the 3D system was determined by taking images of the spheroids and determining the aspect ratio of the spheroid using the ImagePro Plus 4.5 software.

### 2.5 Real-time PCR

Total RNA was extracted from cells cultured in 2D or in 3D using the Total RNA Purification Kit (Norgen Biotek Corp, Thorold, ON, Canada) according to the manufacturer’s instructions. RNA concentrations were determined using the NanoDrop-One 1000 spectrophotometer (Thermo Scientific, Waltham, MA, USA). Three μg of total RNA were reverse transcribed using the FIREScript RT cDNA synthesis Mix with oligo (dT) and random primers kit (Solis BioDyne, Tartu, Estonia) according to manufacturer’s instructions. Eighty ng of the resulting cDNA were amplified in the StepOne system (Applied Biosystems, Foster City, CA, USA) in triplicates, using the 5X HOT FIREPol EvaGreen qPCR Mix Plus (Solis BioDyne) and 200nM of the primers (**Table 1**). We used initial activation at 95°C for 12 min followed by 40 cycles of 95°C for 15 sec, 60°C for 20 sec, and 72°C for 20 sec., to determine the mRNA expression level of the different genes or their endogenous reference gene PBGD. Alternatively, 1μg of total RNA were transcribed using the microRNA cDNA synthesis kit (QuantaBio, Beverly, MA, USA) and then the universal primer and the miR-146a-5p or the RNU6B (U6) small RNA that served as endogenous control were used as primers (**Table 1**) and amplified in triplicates using the Syber green super mix (QuantaBio) kit. The relative levels of gene expression were calculated by the comparative ^ΔΔ^CT method, using the non-treated cells as calibrators in each experiment, to compare the relative quantity (RQ) between the samples.

**Table 1:** list of primers used for qPCR amplification.

Gene amplified	Forward primer	Reversed primer
*EMMPRIN*	5’-TGGCCTTCACGCTCTTGAG	5’-CAACGCCACTGCTGTTCAAA
*Snail*	5’-TGTCTGCACGACCTGTGGAAAG	5’-CTTCACATCCGAGTGGGTTTGG
*Slug*	5’-TCTGTGGCAAGGCTTTCTCCAG	5’-TGCAGATGTGCCCTCAGGTTTG
*Twist1*	5’-GATTCAGACCCTCAAACTGGCG	5’-AGACGGAGAAGGCGTAGCTGAG
*Zeb1*	5’-ATTCAGCTACTGTGAGCCCTGC	5’-CATTCTGGTCCTCCACAGTGGA
*SOX2*	5’-AACGGCAGCTACAGCATGATGC	5’-CGAGCTGGTCATGGAGTTGTAC
*NR2F1*	5’-CCAACAGGAACTGTCCCATCGA	5’-CCGTTTGTGAGTGCATACTGGC
*p21*	5’-TCGCTGTCTTGCACTCTGGTGT	5’-CCAATCTGCGCTTGGAGTGATAG
*PBGD*	5’-CAGTTTGAAATCATTGCTATGTCCA	5’-CTCCAATCTTAGAGAGTGCAGTATC
*miR-146a-5p*	5’-TGAGAACTGAATTCCATGGGTTA	
*RNU6B (U6)*	5’-GCAAATTCGTGAAGCGTTCC	
*Universal primer*		5’-GCATAGACCTGAATGGCGGTA

### 2.6 Sandwich ELISA

Concentrations of the mouse EMMPRIN in cell supernatants (diluted 1:100) were determined using the matched antibody pair kit (Abcam), and those of MMP-9 and VEGF (supernatants diluted 1:100) were evaluated with the DuoSet ELISA kits (R&D Systems, Minneapolis, MN). Phosphorylation of the ERK1/2 and the p38 MAPKs in cell lysates (R&D systems) as well as of EMMPRIN (Abcam) in cell lysates were assessed using their DuoSet ELISA kits and normalized to the total protein. All kits were carried out according to the instructions of their manufacturers.

### 2.7 Wound assay

The bEND3 mouse microvessel endothelial cells were plated in a 96-well plate (4x10^4^ cells/well/100 μl) in full medium and allowed to reach confluency overnight. A scratch was made with a pipette tip, and detached cells were washed away. Then, conditioned media (CM) obtained from previous experiments with CT26 cells cultured in 2D or in 3D were diluted 1:2 with the full endothelial cell medium and applied onto the scratched bEND3 cells. Images of the scratch at the beginning of the experiment (time 0h) and at the end of the experiment (time 24h) were obtained by an inverted microscope. The length to which cells migrated to was measured using the ImagePro Plus 4.5 software.

### 2.8 Invasion assay

The upper chamber of inserts (8μM pores size), was coated with 50μL of 0.15 mg/ml liquefied basement membrane extract (Coultrex^®^ BME, R&D systems), and incubated at 37°C for 4 hours to solidify the BME. Then, excess fluid was discarded, and the ECM was equilibrated with 40μL of medium for 1 hour. CT26-WT, CT26-KD or CT26-NC cells (3x10^4^ cells/insert) were seeded as monolayers in the upper chamber in 100μL serum starvation medium. Alternatively, formed spheroids were transferred to the upper chamber of the inserts. The inserts were then transferred to a 24-well plate, where the lower chamber was filled with 500μL of medium with 10% FCS as a chemoattractant. Cells were allowed to migrate to the other side of the insert membrane for 24 h, and were then fixed in 4% formaldehyde for 15 minutes. Cells or spheroids in the upper side of the insert were thoroughly removed using a cotton swab, and cells in the lower part of the membrane were stained with 0.05% Crystal Violet for 20 min. at room temperature. Images of three separate fields were taken of each membrane (Magnification x10). The stained area of the cells that migrated to the other side of the membrane was evaluated using the ImagePro Plus 4.5 software and the area of cells migrating towards serum starvation medium was subtracted from it.

### 2.9 Immunofluorescence

CT26-WT, CT26-KD or CT26-NC cells (3x10^4^ cells/100μl full medium) were seeded on sterile cover slips and allowed to adhere overnight, after which the medium was replaced with serum starvation medium, and cells were incubated for 48 h. Then, cells were fixed with 300μL of 4% formaldehyde for 10 min following by three washes with PBS. Cells were then blocked in buffer (2% donkey normal serum, 0.1% Triton-X100 in PBS) for 1 hour at room temperature. The primary antibodies rabbit anti-Vimentin (diluted 1:700, Abcam) or rat anti-E-cadherin (diluted 1:200, BioLegend, San Diego, CA, USA) were added and incubated in 120μL of the blocking buffer overnight at 4°C. Next, cells were washed with PBS three times, and the secondary antibodies donkey anti-rabbit Alexa Fluor^®^ 555 (diluted 1:1000) or donkey anti-rat Alexa Fluor^®^ 488 (diluted 1:1000) were incubated for 1 h in the dark at room temperature. Cells were washed again once in PBS, and once with PBS with 10nM DAPI for 5 min. Coverslips were mounted with Fluoromount G on carrying glass, and sealed with a nail polish.

### 2.10 Immunohistochemistry

Spheroids were formalin fixed and paraffin embedded (FFPE), and then cut into 4μm thick sections. The sections were deparaffinized, with K-clear plus (Kaltek, Saonara, Italy), and rehydrated with decreasing ethanol concentrations. Antigen retrieval was carried out for 15 min in a microwave in citrate buffer pH 6.0, and endogenous peroxidase activity was blocked with 10 min incubation with 3% hydrogen peroxide. Slides were then blocked with 4% BSA with 0.02% triton X100 and incubated at 4°C overnight with the rabbit anti-vimentin antibody diluted 1:1400 (Abcam). After three washes (0.05% Tween 20 in PBS), the HRP-polymer anti-rabbit (Zytomed, Berlin, Germany) was added and incubated for 1 h at room temperature, following three washes and incubation for 40 min with the DAB substrate kit (Zytomed). Sections were counterstained with hematoxylin Gil III (Leica Biosystems, Deer Park, IL) and were imaged in the bright field trinocular microscope (Olympus BX-60, Tokyo, Japan) and acquired with the MS60 camera and the MShot Image Analysis System V1 (MSHOT, Guangzhou Micro-shot Technology Co., Guangzhou, China).

### 2.11 Flow cytometry

CT26-WT, CT26-KD and CT26-NC cells (10^6^ cells each) were centrifuged and re-suspended in PBS with 1% FCS, and then incubated with 0.5µg of rat anti-mouse FITC-conjugated anti-EMMPRIN or with its isotype control (BioLegend) for 30 min at 4°C. After washing, the cells were fixed in 0.1% formaldehyde and analyzed by flow cytometer, (LSRFortessa, BD Biosciences, San Jose, CA). Dead cells were excluded from the analysis by their forward and sideway light-scattering properties.

### 2.12 Western blot

Equal amounts of cellular lysates (20 μg/lane) that were extracted in RIPA buffer were loaded on a gradient 4-20% SDS-PAGE, and proteins were separated by electrophoresis and transferred onto a nitrocellulose membrane (Advansta, San Jose, CA, USA). Block-Chemi (Advansta) reagent served to block the membranes for 1 hour. Then the primary antibodies goat anti-EMMPRIN (R&D systems, diluted 1:1000), mouse anti-TRAF6 antibody (Santa Cruz Biotechnology, Dallas, TX, USA, diluted 1:500) or mouse anti-β-actin (ProteinTech, Rosemont, IL, USA, diluted 1:10,000) in blocking solution were added for overnight incubation at 4°C, following 3 washes in TBST buffer (1xTris-buffered saline with 0.1% Tween 20). Then the HRP-conjugated secondary antibodies donkey anti-goat IgG or goat-anti-mouse IgG (Jackson ImmunoResearch Labs, West Grove, PA, USA, diluted 1:5,000) was incubated for 1 hour, and after three more washes, the membranes were incubated with the WesternBright ECL HRP substrate (Advansta). The protein bands were visualized using the Omega Lum G imaging system (Aplegen, Pleasanton, CA, USA).

### 2.13 Reverse transfection with miR-146-5p mimic

The Lipofectamine RNAi MAX (Thermo Fisher Scientific/Ambion, Austin, TX, USA) was diluted 1:25 in Opti-MEM medium and combined with an Opti-MEM medium containing 150 nM of miRNA-146a-5p mimic (final concentration was 30 nM), or its negative controls (Thermo Fisher Scientific/Ambion). The generated lipid complexes were spread on a well in a 24-well plate for 20 minutes. Then the CT26-WT cells (8x10^4^ cells) were added in full medium without antibiotics. After 24 hours of incubation, cells in monolayers were washed with PBS and starvation medium (300 μl) was added for 48 hours. To generate transfected spheroids, cells were trypsinized and 5x10^3^ cells were plated in a well of 96-well plates coated with 1% agarose in full medium (70μl) for 48 hours. At the end of the incubation cellular lysates, RNA extracts or supernatants were collected for analysis.

### 2.14 3D metastatic outbreak model on a basement membrane extract system

To simulate metastatic outbreak that relies on the proliferation of metastatic cells in a 3D matrix, we followed the protocol described in (38). Briefly, we coated 96-well plates with 40μl of ice cold Coultrex™, that was solidified by placing the plates in a humidified incubator for 30 min. Cells were then seeded onto the BME-coated plates (10^3^ cells/well/60μl) in CT26 serum-starvation medium supplemented with 2% FCS + 2% BME (assay media). Every 4 days, cells were re-fed with the assay media. Images were taken at different time points. The mean area of the resulting cell structures was quantified using ImagePro Plus 4.5 software.

### 2.15 Statistical analysis

All experiments were independently repeated at least four times in triplicates, and results are represented as mean ± standard error of mean (SEM). Statistical significance between three groups was determined using one-way ANOVA followed by Bonferroni’s post-hoc multiple comparison test. Statistical significance was determined at P-values less than 0.05 (α<0.05). All statistical analyses were performed using Prism 10.10 software (GraphPad, La Jolla, CA, USA).

## 3 Results

### 3.1 EMMPRIN-KD cells have reduced EMMPRIN expression

To study the involvement of EMMPRIN in the formation of spheroids and in their properties, we stably knocked-down its expression in the mouse colon carcinoma cell line CT26, as described in the methods section and in (36). The expression of EMMPRIN was then validated at the RNA and protein levels, and as a secreted protein. Although the levels of EMMPRIN mRNA were not different between the CT26-WT, CT26-KD and CT26-NC cells (**Figure 1A**), the protein levels at the membrane (**Figure 1B**), in the cells lysates (**Figure 1C**) and the secreted levels (**Figure 1D**) were significantly reduced by about 50-60%. This reduction was also confirmed in western blot analyses (**Figures 1E, F**). This reduced level of expression in the CT26-KD cells still allowed their proliferation and survival. The lack of change in the EMMPRIN mRNA levels suggests a post-translational regulation of EMMPRIN, and we have previously implicated miR-146a-5p in such a regulation (39).

### 3.2 Reduced EMMPRIN expression changes cell morphology

**Figure 2:**
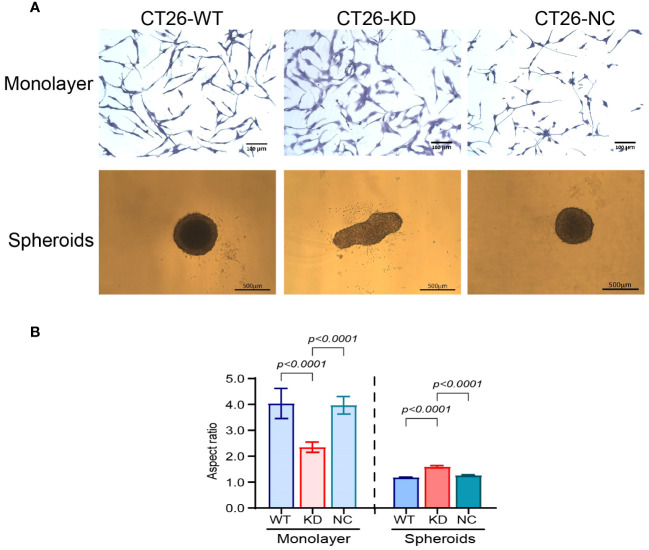
Reduced EMMPRIN expression changes cell morphology. **(A**, upper panel) Representative images of CT26-WT, CT26-KD and CT26-NC cells that were seeded (3x10^3^ cells/well/100μL) as monolayers. After 48 h of incubation in serum-starvation medium, cells were stained with 0.1% crystal violet, bar size 100μm. CT26-KD cells are more spread and adhered to the culture dish compared with CT26-WT and CT26-NC. **(A**, lower panel) Representative images of the cells seeded as spheroids (5x10^3^ cells/well/100μL) on 1% agarose. Cells were allowed to grow for 3 days. Bar size 500μm. CT26-KD form elongated aggregates, in contrast to the spheres generated by CT26-WT and CT26-NC cells. **(B)** Analysis of the aspect ratio of cells seeded as monolayers or spheroids (n=6 for monolayers, n=48 for spheroids). Data are presented as means ± SEM, and analyzed by one-way ANOVA followed by Bonferroni’s post-hoc test.

The morphology of the cells changed both in monolayers and in spheroids. CT26-WT and CT26-NC monolayers presented a network of elongated cells, indicating their mesenchymal state, whereas the CT26-KD cells were a little more spread on the culture dish (**Figure 2A**, upper panel). The change in the morphology was reflected by the high aspect ratio (4.04 ± 0.5) that describes the spindle-like morphology of the CT26-WT and CT26-NC cells, which was reduced by 42% in the CT26-KD cells (**Figure 2B**). Similar results were obtained for the mouse mammary carcinoma 4T1 cells, where the aspect ratio was decreased in the 4T1-KD cells by 18.5%, and for the Lewis lung carcinoma (LLC) cells where the aspect ratio was decreased by 27.5% in the LLC-KD cells (**Supplementary Figures S1A, B**).

In the 3D model, the CT26-WT and CT26-NC cells formed round, almost perfect spheres when plated on wells coated with 1% agarose, which was manifested by the aspect ratio of 1.18 ± 0.016. These spheroids were large, with a mean diameter of 573 ± 12.56μm (**Figure 2A**, lower panel). Similarly, the 4T1-WT and the LLC-WT cells also generated round spheroids with an aspect ratio of 1.054 ± 0.005 and 1.046 ± 0.004μm, respectively (**Supplementary Figures S1A, B**). In contrast, the CT26-KD cells formed non-spherical and more elliptic aggregates (mean of long diameter 706 ± 22, mean of short diameter 416 ± 10.1), resulting in an increase of their aspect ratio by 37%. However, despite the different morphology, the CT26-WT, CT26-KD and CT26-NC cells did not present differences in their area (253,000 ± 11,325 μm), suggesting that their cellular mass was not different. Likewise the 4T1-KD cells and the LLC-KD cells generated non-spherical elliptic aggregates, with an aspect ratio of 1.78 ± 0.17 and 1.42 ± 0.004, respectively (**Supplementary Figures S1A, B**).

### 3.3 Reduced EMMPRIN expression and 3D organization as spheroids promote the mesenchymal phenotype and dormancy

To explore the effects of the spatial organization on the EMT status of CT26 cells that express EMMPRIN in high (CT26-WT, CT26-NC cells) or low levels (CT26-KD cells), we evaluated some of the properties that are associated with a mesenchymal phenotype. These include their proliferative capacity, drug resistance, invasiveness, angiogenic potential, and ability to express genes related to the EMT process and to dormancy. However, because monolayers and spheroids were cultured in different conditions, we compared separately the magnitude of the difference between the CT26-WT/CT26-NC and CT26-KD cells in each of the different spatial organizations.

**Figure 3:**
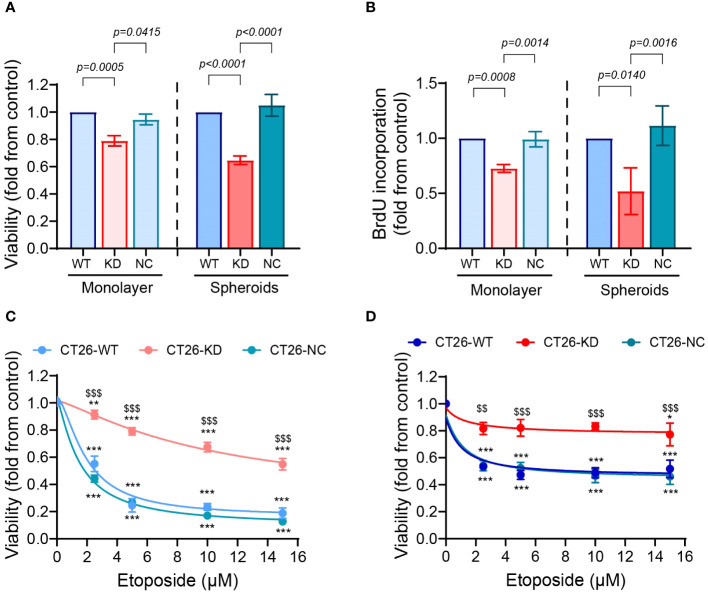
Reduced EMMPRIN expression inhibits proliferation and enhances drug resistance. CT26-WT, CT26-KD and CT26-NC were seeded as monolayers (10^4^ cells/well/100μL) or as spheroids (5x10^3^ cells/well/100μL), and incubated for 48 hours or 72 hours, respectively. Proliferation of the cells was evaluated by **(A)** the CCK-8 kit (n=9 for monolayers and for spheroids) and by **(B)** the incorporation of BrdU into the DNA of proliferating cells (n=7 for monolayers, n=4 for spheroids). Data are presented as means ± SEM, and analyzed by one-way ANOVA followed by Bonferroni’s post-hoc test. **(C, D)** Cells were incubated for 48 h as monolayers or as spheroids with increasing concentrations of etoposide as indicated (0-15μM), and the viability of the cells was determined using the CCK-8 kit assay (n=5 for both monolayers and spheroids). Data are presented as means ± SEM, and analyzed by two-way ANOVA followed by Bonferroni’s post-hoc test. **, p<0.01, ***, p<0.001 relative to no addition of etoposide, $$, p<0.01, $$$, p<0.001 relative to the other groups at the same concentration.

The CT26-KD cells cultured as monolayers and as spheroids presented reduced rates of proliferation relative to their respective CT26-WT and CT26-NC cells, as assessed both by the cell counting kit (CCK-8) and by the DNA incorporation of BrdU (**Figures 3A, B**). In the monolayers, CT26-KD cells showed a 21% or 27% reduction relative to the CT26-WT cells in the CCK-8 and BrdU assays, respectively, whereas in the spheroids the proliferation was inhibited by 35% and 48%, respectively. Thus, in cells with reduced EMMPRIN expression, proliferation in spheroids is attenuated more than in monolayers.

To assess the ability of cells to develop drug resistance, we exposed them to the chemotherapeutic drug etoposide, an inhibitor of topoisomerase II that is highly expressed in CT26 cells (40). Only about 50% of both the CT26-WT and CT26-NC cells cultured as monolayers survived upon exposure to low concentrations of etoposide, reaching 18% and 13% survival in the high concentration of 15μM etoposide, respectively (**Figure 3C**). In contrast, the CT26-KD cells in monolayers exhibited greater resistance to the drug, reaching 55% survival at 15μM etoposide. The 3D organization in spheroids conferred resistance to the drug even in the CT26-WT and CT26-NC cells, which exhibited 52% and 46% survival at 15μM etoposide, respectively, consistent with previous reports of increased drug resistance in spheroids relative to monolayers (41). The CT26-KD cells in spheroids exhibited 77.3% survival at that concentration (**Figure 3D**). Thus, the reduced EMMPRIN expression together with the spheroid structure conveyed the best protection against etoposide-induced cell death.

**Figure 4:**
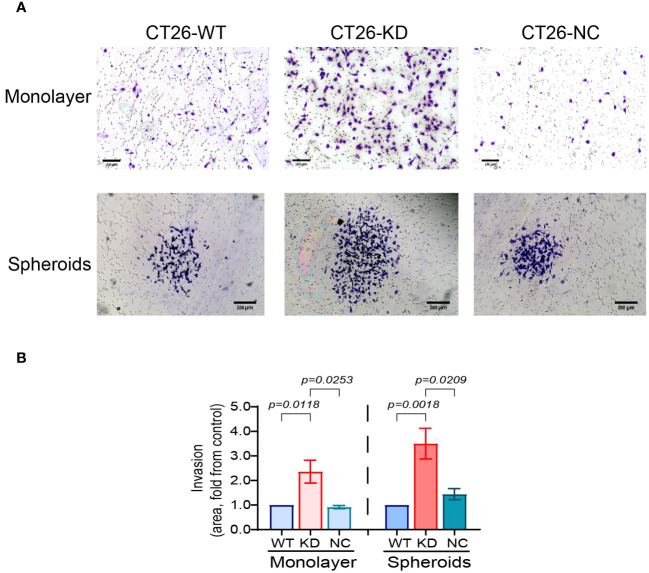
Reduced EMMPRIN expression elevates cell invasiveness. Parental CT26-WT, CT26-KD and CT26-NC cells were seeded (3x10^4^ cells/well/100μL) as monolayers in the upper chamber of a Boyden chamber membrane (pore size 8μn) coated with Coultrex^®^ basement membrane extract (BME), and allowed to migrate to the other side of the insert membrane for 24 h and then fixed and stained with 0.05% Crystal Violet. Alternatively, cells were seeded (5x10^3^ cell/well/100μL) on 1% agarose and after 3 days, spheroids were transferred into the upper chamber of the insert. **(A)** Representative images, bar size for monolayer is 150μm for monolayers and 200 μm for spheroids, and **(B)** their quantitation (n=6 for monolayers, n=5 for spheroids). Data are presented as means ± SEM, and analyzed by one-way ANOVA followed by Bonferroni’s post-hoc test.

Invasiveness is one of the hallmarks of EMT and a mesenchymal phenotype. To explore the invasive abilities of cells, CT26-WT, CT-KD and CT26-NC cells were seeded as monolayers or as spheroids in inserts coated with basement membrane extract (BME), and the area of the cells that migrated and invaded through the extract to the lower side of the insert membrane was evaluated. Relative to their parental CT26-WT or to CT26-NC cells, CT26-KD cells that were seeded as monolayers showed a 2.4-fold increase in their ability to invade the Coultrex^®^-coated membrane. CT26-KD cells that were seeded as spheroids exhibited a 3.5-fold increase in their invasive abilities compared to their CT26-WT or CT26-NC counterparts (**Figures 4A, B**).

**Figure 5:**
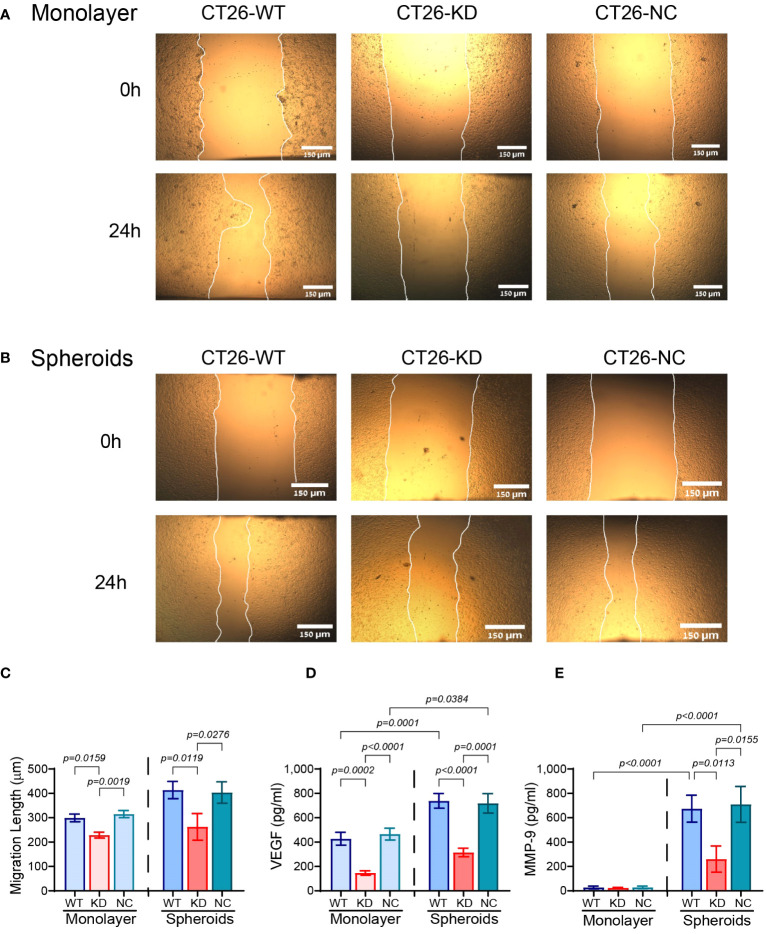
Reduced EMMPRIN expression inhibits the angiogenic potential. **(A)** CT26-WT, CT26-KD and CT26-NC cells were seeded in serum starvation medium (8x10^4^ cells/well/300μL) in 24-well plates for 48 h, their supernatants were collected, diluted (1:2) and applied onto a scratched monolayer of the bEND3 mouse endothelial cells. **(B)** Alternatively, CT26-WT, CT26-KD and CT26-NC cells (5x10^3^ cells/well/100μL) were seeded in 96-well plates coated with 1% agarose, allowed to form spheroids for 3 days, and their collected supernatants were used in the same manner on bEND3 cells. **(A, B)** Representative images were taken at the start of the experiment (0h) and after 24 hours (24h). Bar size for monolayers and spheroids is 150μM. **(C)** The distance that the cells migrated to in order to close the gap was measured (n=7 for monolayers, n=8 for spheroids). The concentrations of **(D)** VEGF (n=7 for monolayers, n=8 for spheroids), and **(E)** MMP-9 (n=7 for monolayers, n=7 for spheroids) were measured by ELISA. Data are presented as means ± SEM, and analyzed by one-way ANOVA followed by Bonferroni’s post-hoc test.

The reduced expression of EMMPRIN also affected different aspects of angiogenesis. The wound assay was carried out using conditioned media (CM) derived from either the monolayers or the spheroids that were diluted (1:2) with full medium, and applied onto a scratched monolayer of the bEND3 mouse endothelial cells. Factors secreted from the monolayer CT26-KD cells resulted in a 23% attenuation in the rate of gap closure relative to CM derived from CT26-WT or CT26-NC cells (**Figures 5A, C**), suggesting that EMMPRIN participates in determining the angiogenic potential. When cells were seeded as spheroids, the attenuation in bEND3 cell migration based on CT26-KD relative to CT26-WT cells was 36.5% (**Figures 5B, C**). We next examined the levels of pro-angiogenic factors in the CM, and detected that the secretion of VEGF in CT26-KD monolayers and spheroids was reduced by 66% and 57% respectively, relative to their respective CT26-WT counterparts (**Figure 5D**). Interestingly, no changes were observed in the secretion of MMP-9 in the monolayer cells, probably due to the lack of any stimulus that induces MMP-9. In contrast, MMP-9 secretion was enhanced by the CT26-WT and CT26-NC spheroids, and the CT26-KD cells exhibited a 61% reduction relative to the CT26-WT spheroids (**Figure 5E**).

**Figure 6:**
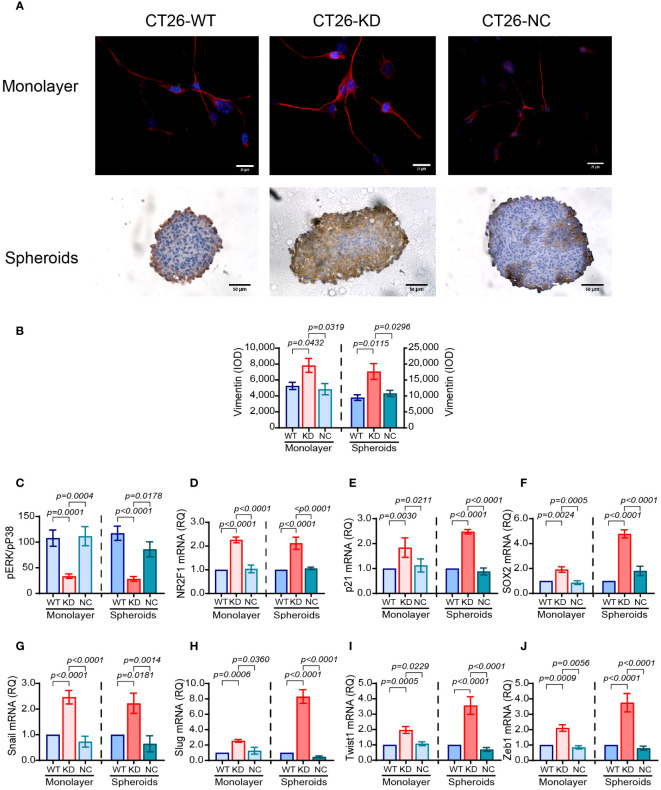
Reduced EMMPRIN expression enhances dormancy. CT26 tumor cells were grown as described before, in monolayers or in spheroids. **(A)** Representative images of staining for the expression of vimentin in monolayers by immunofluorescence (bar size is 25μm) and in spheroids by immunohistochemistry (bar size is 50μm). **(B)** Quantitation of vimentin expression (n=7 for monolayers, n=8 for spheroids). **(C)** Lysates were extracted from the cells, and the concentrations of phosphorylated p38 and ERK1/2 were determined by ELISA. The ratio pERK/pP38 was calculated (n=6 for both monolayers and spheroids). **(D-J)** Total RNA was extracted from monolayers or spheroids, transcribed to cDNA and amplified using primers for EMT-TFs or dormancy markers (**Table 1**). **(D)** NR2F1 (n=5 for monolayers and spheroids). **(E)** p21 (n=4 for monolayers and spheroids). **(F)** SOX2 (n=6 for monolayers and spheroids). **(G)** Snail (n=5 for monolayers and spheroids). **(H)** Slug (n=5 for monolayers and spheroids). **(I)** Twist1 (n=4 for monolayers and spheroids). **(J)** Zeb1 (n=4-5 for monolayers and spheroids). Data are presented as means ± SEM, and analyzed by one-way ANOVA followed by Bonferroni’s post-hoc test.

The reduced expression of E-cadherin and the increase in the expression of vimentin is a hallmark of epithelial cells undergoing EMT to acquire a more mesenchymal phenotype. CT26 cells do not express E-cadherin (40), and we have validated this by immunofluorescence (data not shown), so they have already acquired a mesenchymal phenotype, as suggested by their morphology. However, relative to their respective CT26-WT cells, CT26-KD cells enhance their vimentin expression by 1.48-folds in monolayers and by 1.86-folds in spheroids (**Figures 6A, B**). Interestingly, the expression of vimentin in the spheroids is located at the rims in CT26-WT and CT26-NC cells, but penetrates towards the core of the spheroids in CT26-KD cells (**Figure 6A**, lower panel). This may suggest that the reduction in EMMPRIN expression drives the cells towards an even more mesenchymal phenotype than their parental CT26-WT cells. The activation of the EMT program in these cells is also evident by the increase in the expression of the EMT-TFs snail, slug, twist1 and zeb1, each by about 2-folds in CT26-KD cells relative to CT26-WT cells seeded as monolayers (**Figures 6G–J**). When the cells were seeded as spheroids, the CT26-KD cells enhanced the expression of snail (2-folds), slug (8-folds), twist1 and zeb1 (3.5-folds) relative to the CT26-WT spheroids (**Figures 6G–J**).

We next asked whether CT26-KD cells are more dormant than their parental CT26-WT cells. The ratio of the phosphorylated kinases ERK1/2 and p38 is often used to assess dormancy, as p38 MAPK can inhibit proliferation and ERK1/2 MAPK can promote proliferation (42). We show that relative to the CT26-WT cells, CT26-KD cells in monolayers exhibit a 69% reduction in the ERK/p38 ratio, and a 76% reduction when seeded as spheroids (**Figure 6C**). Thus, phosphorylation of ERK1/2 is reduced and phosphorylation of p38 is increased, suggesting enhanced dormancy in CT26-KD cells. Likewise, the gene expression of the dormancy markers NR2F1 and p21 in CT26-KD cells was enhanced by about 2-folds in both monolayers and spheroids, and the stemness marker SOX2 mRNA was enhanced by 2-folds in monolayers and by 4.7-folds in spheroids, relative to their respective CT26-WT cells (**Figures 6D–F**).

### 3.4 Reduced EMMPRIN prevents a metastatic outbreak

**Figure 7:**
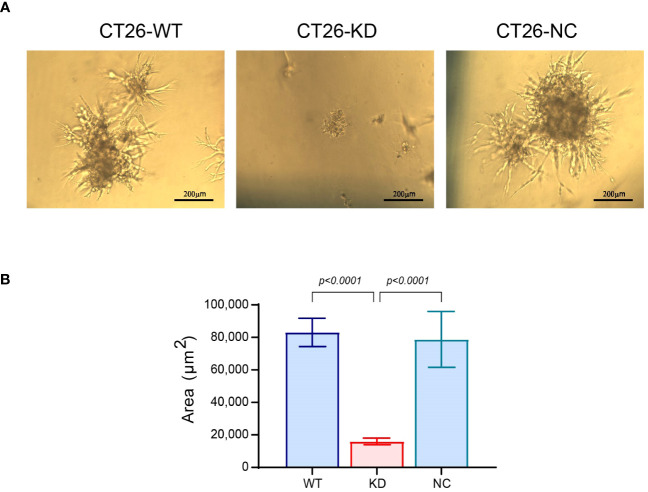
Reduced EMMPRIN expression prevents the metastatic outbreak. Wells in 96-well plates were coated with Coultrex^®^ basement membrane extract (40μl/well) that was solidified by 30 min incubation at 37°C. CT26 cells (10^3^ cells/well) were seeded in 60μl of medium with 2% BME and 2% FCS. Every 3-4 days the cells were re-fed with 30μl of the assay medium, and cells were incubated for a total of 13 days. **(A)** Images were taken at the end of the incubation (bar size 200μm), and **(B)** the area of the cell aggregates was determined (n=35). Data are presented as means ± SEM, and analyzed by one-way ANOVA followed by Bonferroni’s post-hoc test.

As CT26-KD cells tend to be more mesenchymal and dormant than their parental CT26-WT cells, we asked whether this could be reflected in a functional assay. We evaluated the potential of the cells to escape dormancy and generate a metastatic-like structure using an *in vitro* system that was previously demonstrated to distinguish between dormant and metastatic cells (38, 43), and is based on the ability of cells to aggregate and form 3D structures. In contrast to spheroids that get more compacted over time and are devoid of contact with ECM proteins, cells in this assay interact with the basement membrane extract (BME), and proliferate over time to generate an irregular 3D structure that resembles a metastatic lesion and simulates the process of the metastatic outbreak. After 13 days in culture, the CT26-WT and CT26-NC cells developed such 3D structures with typical protrusions and a mean area of 83,090 ± 8,730 μm^2^, thus mimicking the metastatic outbreak. In contrast, the CT26-KD cells remained as very small cellular aggregates, and their mean area was smaller by 81% (**Figure 7**). Thus, EMMPRIN expression is required for the process of the metastatic outbreak. These results were also confirmed in LLC-WT cells that generated metastatic-like lesions with an average area 143,647 ± 24,810 μm^2^, compared to the LLC-KD cells that did not generate such structures and their average area was reduced by 98% (**Supplementary Figure S1C**).

### 3.5 EMMPRIN and miR-146a-5p form a negative feedback loop

**Figure 8:**
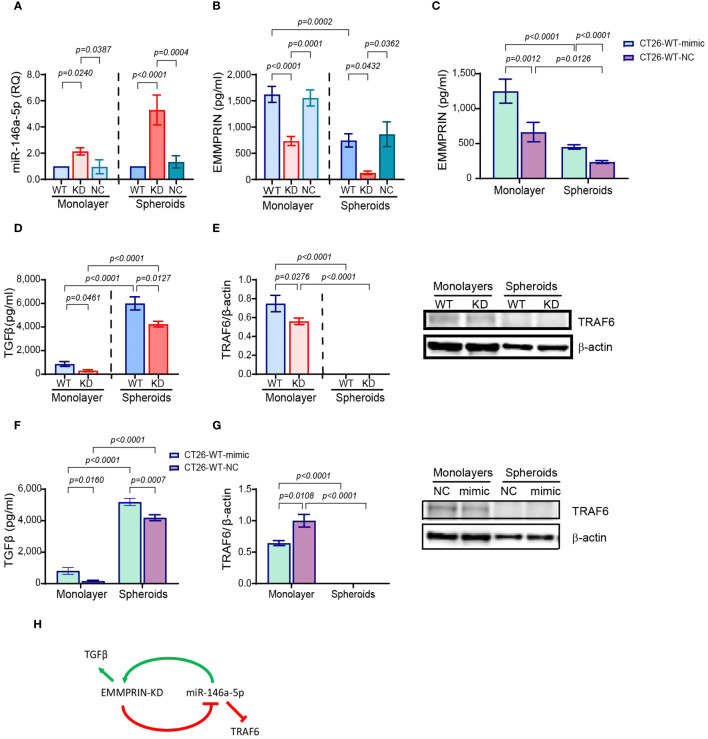
EMMPRIN and miR-146a-5p form a negative feedback loop. CT26-WT, CT26-KD and CT26-NC cells were seeded as monolayers (8x10^4^/300μl) in serum starvation medium for 48 hours, or as spheroids (5x10^3^/100μl) in full medium on 1% agarose-coated wells for 3 days. Total RNA was extracted from the cells and supernatants were collected. **(A)** Expression of miR-146-5p normalized to CT26-WT, and accumulation of **(B)** EMMPRIN (n=6-8) and **(D)** TGFβ (n=6), **(E)** protein expression of TRAF6 was determined by western blot analysis (n=4-6). Additionally, CT26-WT cells were transfected with the miR-146a-5p mimic or its negative control (NC), supernatants were collected and protein lysates were extracted. The concentrations of **(C)** EMMPRIN (n=7) and **(F)** TGFβ (n=7) were determined, and **(G)** TRAF6 expression was measured by western blot analysis (n=5). Data are presented as means ± SEM, and analyzed by one-way ANOVA followed by Bonferroni’s post-hoc test. **(H)** A model of the negative feedback loop between miR-146a-5p and EMMPRIN.

We have demonstrated that EMMPRIN affects cell morphology, promotes the mesenchymal phenotype and prevents dormancy in spheroids more than in monolayer, raising the question what determines the differences in EMMPRIN expression. Since we have previously linked miR-146a-5p to post-transcriptional regulation on EMMPRIN expression (39), we next evaluated the expression of miR-146a-5p in monolayers and spheroids. Relative to their respective CT26-WT cells, the expression levels of miR-146a-5p were increased in CT26-KD cells by 2-folds in monolayers, and by 5.3-folds in spheroids (**Figure 8A**), whereas the accumulated levels of EMMPRIN in the supernatants were decreased in CT26-KD cells by 55% in the monolayers and by 82% in the spheroids (**Figure 8B**). To reconfirm the link between miR-146a-5p and EMMPRIN and to simulate miR-146a-5p overexpression as observed in the CT26-KD cells, we transfected CT26-WT cells with the miR-146a-5p mimic (CT26-WT-mimic) or its negative control (CT26-WT-NC). Relative to the negative control, the accumulation of EMMPRIN in the supernatants was increased by 1.9-folds in both monolayers and spheroids (**Figure 8C**). Thus, miR-146a-5p positively regulates EMMPRIN and enhances its secretion, whereas EMMPRIN inhibits the levels of miR-146a-5p, generating a negative feedback loop (**Figure 8H**).

We next reasoned that in a negative feedback loop, proteins that are targeted by either miR-146a-5p or EMMPRIN should be affected by the manipulation of any of the regulators. Therefore, we looked first at the secretion of transforming growth factor beta 1 (TGFβ1), that is regulated by EMMPRIN, but not by miR-146a (44, 45). Relative to CT26-WT cells, TGFβ was reduced in CT26-KD cells both in monolayers and in spheroids (**Figure 8D**). Conversely, TGFβ was increased in CT26-WT-mimic cells relative to CT26-WT-NC cells (**Figure 8F**). The tumor necrosis factor receptor−associated factor 6 (TRAF6) is a verified target of miR-146a-5p (46), but not of EMMPRIN. TRAF6 expression was reduced in CT26-KD in monolayers relative to the CT26-WT cells (**Figure 8E**), and in the CT26-WT-mimic cells seeded as monolayers relative to the CT26-WT-NC cells (**Figure 8G**). However, no TRAF6 protein expression could be detected in any of the cells when seeded as spheroids (**Figures 8E, G**).

## 4 Discussion

In this study, we demonstrate that EMMPRIN is an important participant in the formation of spheroids as a spatial organization of tumor cells, and that this organization and the expression of high levels of EMMPRIN promote cellular properties that enhance their ability to metastasize. Spheroids have emerged as a useful tool that more closely simulates the *in vivo* organization of solid tumors in comparison to the standard *in vitro* culturing approach of monolayers, and provide a better *in vitro* platform to study the enhanced metastatic properties of tumor cells (19). We emphasize that in our study the cells seeded as monolayers or spheroids were not stimulated in any other way, allowing us to assess the effects of the spatial organization alone. However, because of differences in the conditions of the cultures (e.g., cell number, plastic dishes vs. agarose-coated dishes, presence of FCS in the spheroids), we did not directly compare monolayers to spheroids, but only the magnitude of change between the CT26-KD and CT26-WT/CT26-NC cells in each condition.

First, we show that the reduced expression of EMMPRIN in the CT26-KD cells changes cell morphology both in 2D monolayers and in 3D spheroids. CT26-KD cells in monolayers spread a little on the dish surface, although they still maintained an elongated mesenchymal appearance. However, when seeded as spheroids, CT26-KD cells formed elliptic structures that were very different from the almost perfect spherical structure generated by the CT26-WT and CT26-NC cells. These phenomena also occurred in the 4T1 and LLC cell lines, indicating a general phenomenon. Spheroids approximately 300–500μm in size have been found to best mimic *in vivo* tumors, specifically regarding the hypoxic zones and proliferative gradients (16). After calibrating the number of cells, our CT26-WT and CT26-NC spheroids, and even the CT26-KD 3D structures, were still within this range. This suggests the existence of similar gradients of hypoxia and nutrients, and rules out size as a cause for the differences in metastatic properties. Thus, EMMPRIN plays a role in determining cell morphology, probably due to its role as an adhesion molecule, or as a hub protein that binds to other adhesion molecules (30). Adherens junctions, and E-cadherin in particular, are considered necessary in maintaining the columnar epithelial structure, and their reduction is associated with the spindle-like, mesenchymal morphology (47). The parental CT26-WT cells that do not express E-cadherin at all (40), exhibit a spindle-like, mesenchymal morphology, and its spheroids generated an almost perfect sphere, suggesting that other adhesion molecules may compensate for the absence of E-cadherin. This spherical structure was lost in the CT26-KD cells, suggesting that EMMPRIN is a protein that may compensate for the absence of E-cadherin, and may have a supportive role in determining the spatial organization of the cells. This is supported by a study demonstrating that cells with high expression levels of EMMPRIN generated more spheroids than low expressing EMMPRIN cells (33).

Along the epithelial-mesenchymal axis, the CT26-KD cells seem to be even more mesenchymal than the already mesenchymal CT26-WT or CT26-NC cells, and their organization into spheroids accentuates this even further. We show that CT26-KD cells in spheroids have reduced proliferation rates and reduced angiogenic potential, but enhanced drug resistance, invasiveness, and elevated expression of vimentin and EMT-TFs, relative to the spheroids of CT26-WT cells, and the magnitude of change is larger than the change observed in CT26-KD cells vs. CT26-WT cells in monolayers. Likewise, we demonstrate that in cells with reduced EMMPRIN, expression of genes involved in dormancy is enhanced in spheroids more than in monolayers. Therefore, EMMPRIN has a regulatory role in promoting and maintaining a hybrid EMT status, which is considered more aggressive (9), and in acting as a gatekeeper that prevents entry into a dormant state. Others (35, 48) and we (36) have already implicated EMMPRIN in driving the EMT process, and we have shown that EMMPRIN prevents dormancy in CT26 monolayers interacting with monocytes (36). However, these studies were carried out in monolayers, whereas here we demonstrate that in spheroids, EMMPRIN has an even bigger role in driving EMT and preventing dormancy.

We have examined multiple parameters that collectively allow us to determine the EMT status of the cells. First, we used two approaches to determine the proliferative rate of the cells, the CCK-8 assay, which is based on the metabolic activity of the mitochondria, and the BrdU assay, which relies on the incorporation of the nucleotide analogue BrdU in the DNA of the dividing cell. In these assays, the reduced expression of EMMPRIN inhibited cell proliferation in both monolayers and spheroids, demonstrating that EMMPRIN is involved in cell proliferation, as have been reported before in many types of cancer cells (49–51). Cells aggregated into spheroids are heterogeneous and consist of a proliferative outer rim and a quiescent middle layer, whereas the core is composed of necrotic cells (16, 18). While we do not, in general, directly compare monolayers to spheroids, we note that the microenvironmental stress and the large fraction of cells that are located in the quiescent layer of the spheroid could explain the reduced proliferation rate of spheroids in general (52), and in the CT26-KD cells in spheroids in particular, compared to the same cells in monolayers.

Spheroids confer a protective effect and increase drug resistance. This is explained by the activation of HIF-1α in the quiescent layer of the spheroids that increases the expression of the drug resistance protein P-glycoprotein (Pg) and of anti-apoptotic genes (18), as well as the acidic microenvironment that protonates several chemotherapeutic drugs and inhibits their uptake by the tumor cells (18). EMMPRIN is also involved in drug resistance, as it enhances the synthesis of hyaluronan, which then forms a protective cap around cancer cells and inhibits the entry of chemotherapeutic drugs, and due to EMMPRIN interaction with CD44 that activates the PI3K signalling pathway and induces survival genes (53, 54). Several studies show that decreased EMMPRIN expression or its silencing results in increased sensitivity to chemotherapeutic drugs (53), and cells expressing high EMMPRIN levels showed increased drug resistance compared to cells with low levels of EMMPRIN expression (33). In contrast, we show here that reduced EMMPRIN expression enhances the resistance to etoposide in monolayer, and even more so in spheroids. We did not investigate the expression of HIF1α, Pg or Bcl-2 in the CT26-KD cells, nor the extent of hyaluronan synthesis, and we cannot rule out their involvement in conferring drug resistance. However, we suggest that at least in our system, the proliferative capacity of the cells outweighs other mechanisms, and is negatively associated with drug resistance, as chemotherapeutic drugs specifically target proliferative cells. The low proliferative capacity of CT26-KD cells, in monolayers and even more so in spheroids, is manifested in higher resistance to etoposide.

The invasion assay evaluates the ability of individual cells to degrade the ECM proteins and migrate through the matrix to the other side of the separating membrane. When seeding spheroids on the matrix, the spatial 3D organization disintegrates, and individual cells invade the matrix. We show that CT26-KD cells were more invasive than the CT26-WT or CT26-NC cells, and the difference between CT26-KD cells relative to their parental CT26-WT cells was greater in spheroids than in monolayers. This is in contrast to studies that show that EMMPRIN promotes invasiveness, that cells expressing high levels of EMMPRIN are more invasive than cells expressing low levels of EMMPRIN (33, 55), or that cells with knocked-down expression of EMMPRIN exhibit reduced invasiveness (56, 57). In these studies, the effect of EMMPRIN on invasiveness could be explained by the ability of EMMPRIN to disrupt the interaction between E-cadherin and β-catenin, which stabilizes E-cadherin at the membrane. EMMPRIN can promote the degradation of E-cadherin and the translocation of β-catenin to the nucleus, and EMMPRIN siRNA inhibited cell migration (58, 59). However, CT26 do not express E-cadherin (40), and loss of E-cadherin expression promotes EMT and invasiveness (60, 61). Therefore, we suggest that CT26-WT cells present high invasiveness *a priory*, and the increased invasion exhibited by CT26-KD cells in monolayers or in spheroids is linked to their enhanced mesenchymal phenotype or to another mechanism yet unidentified.

The ability of tumor cells to trigger angiogenesis is critical for their survival, and dormant micro-metastases must induce angiogenesis when they escape dormancy and become macro-metastatic lesions (62). We show here that in CT26-KD cells, the angiogenic potential of both monolayers and spheroids was reduced, as observed by the wound assay and the decrease in VEGF and MMP-9 levels relative to their CT26-WT controls. This coincides with our previous report (36), and other studies (63, 64). Of note, in the wound assay and VEGF secretion, the magnitude of change between CT26-WT and CT26-KD cells in 2D and 3D was similar. In contrast, MMP-9 was not induced in monolayers that received no stimulus, and the spatial organization into spheroids was sufficient to induce MMP-9 secretion, which was reduced in CT26-KD spheroids. The 3D structure of spheroids that induces hypoxia and the HIF-1 transcription factor may contribute to the difference between spheroids and monolayers (18).

The expression of vimentin and the loss of E-cadherin in CT26 cells determines their EMT status as mesenchymal cells, even before EMMPRIN is knocked-down. In CT26-KD cells relative to CT26-WT, the magnitude of change in vimentin expression increases more in spheroids than in monolayers. Similarly, the increase in the expression of the EMT-TFs in CT26-KD cells relative to CT26-WT in spheroids is larger than in monolayers, suggesting that the 3D organization pushes the CT26-KD cells even further towards the mesenchymal end. Thus, EMMPRIN may have a role in keeping the cells in a hybrid epithelial/mesenchymal state that contributes to the aggressiveness of the cells (9, 40).

CT26-KD cells are also more dormant than the CT26-WT or CT26-NC cells. This was evident by the reduction in the pERK/pP38 ratio, that implies that phosphorylated p38 is involved in the inhibited proliferation of these cells (42, 65). Furthermore, the NR2F1, p21 and SOX2 genes that mediate dormancy were enhanced in CT26-KD relative to CT26-WT cells, and spheroids exhibited a greater magnitude of change in p21 and SOX2 gene expression than monolayers. This is in accordance with the ability of cells to co-express markers of dormancy, stemness and EMT simultaneously, as these processes are linked (66).

We also looked at the potential of cells to escape from dormancy and proliferate to form a metastatic-like lesion. Spheroids formation relies on the high hydrophobicity of the agarose that prevents cell interactions with the surface and generates a scaffold-free structure (18), and the intercellular interactions may be maintained by the ability of cells in spheroids to secrete more ECM proteins than monolayers (18). In contrast, during the 3D metastatic outbreak assay, the basement membrane proteins in the extract, that forms a natural scaffold-based hydrogel, allow ECM-cell interactions. Proteins such as collagens, laminin, or fibronectin, can bind to their integrin receptors, activating different signalling pathways (e.g., FAK, ERK, and PI3K pathways) that are linked to proliferation and viability (18, 67). Thus, the 3D metastatic outbreak assay can provide a more accurate *in vitro* tool than spheroids to model the ability of cells to escape dormancy, as it models both the proliferative and invasive properties of the cells. We show that in contrast to the CT26-WT and CT26-NC cells, CT26-KD cells did not form such metastatic-like structures and remained as small aggregates. The binding of EMMPRIN to the β1 integrins, especially α3β1 and α6β1 integrins, was demonstrated to mediate proliferative signals (68, 69). Thus, reduced EMMPRIN expression in the CT26-KD cells and the lack of integrin-mediated interactions with the ECM proteins may be limiting the ability of cells to outbreak from dormancy, as observed in the 3D metastatic outbreak assay. However, a more detailed investigation into the EMMPRIN-integrin interactions is required to determine this.

As we have implicated EMMPRIN in the change of morphology, in promoting EMT and in preventing dormancy, we asked how its expression is regulated in monolayers and spheroids. First, we established that a negative feedback loop exists between EMMPRIN and miR-146a-5p, where miR-146a-5p enhances EMMPRIN expression and EMMPRIN reduces miR-146a-5p levels. We (39) and others (70) have previously identified EMMPRIN as a target of miR-146a-5p regulation. The finding that miR-146a-5p enhances EMMPRIN is consistent with our previous findings and suggests that the regulation of miR-146a-5p on EMMPRIN may be indirect, involving yet another protein that may represses EMMPRIN expression and may be reduced by miR-146a-5p activity. The increase in EMMPRIN secretion in CT26-WT cells that were transfected with the miR-146a-5p mimic also supports this. On the other hand, we now show for the first time that reduced EMMPRIN expression in CT26-KD cells enhances the expression levels of miR-146a-5p, suggesting that EMMPRIN inhibits the expression of this microRNA. Thus, we suggest that a negative feedback loop exists between EMMPRIN and miR-146a-5p that keeps the system within tolerance limits.

The finding of a negative feedback loop suggests that proteins that are targeted by miR-146a-5p could also be affected by EMMPRIN, and vice versa, proteins regulated by EMMPRIN should be affected by miR-146a-5p. Although Smad4 that mediates TGFβ signaling pathway is regulated by miR-146a-5p, TGFβ itself is not regulated by this microRNA, whereas EMMPRIN can regulate it via β-catenin (44, 45), and we show that reduced EMMPRIN expression reduced TGFβ accumulation in the supernatants, as expected. However, overexpression of miR-146a-5p in mimic transfected cells resulted in increased accumulation of TGFβ, suggesting that miR-146a-5p increases EMMPRIN that in turn enhances TGFβ. Moreover, it also provides a mechanistic explanation to the link previously found between miR-146a-5p and the EMT process (71). Conversely, TRAF6 is a well-known target of miR-146a-5p but not of EMMPRIN, and therefore its reduced expression in CT26-WT-mimic cells relative to the negative control is of no surprise. However, in the CT26-KD cells TRAF6 expression was reduced, suggesting that reduced EMMPRIN expression enhanced miR-146a-5p levels, which in turn inhibited TRAF6.

The difference between the CT26-WT cells seeded in monolayers or spheroids could largely be explained by the hypoxic gradient that is formed only in spheroids. Hypoxia can variably affect miR-146a-5p expression levels, and in some cases it has decreased its levels (72, 73). In our system we did not compare the monolayers to spheroids directly, however we did notice an increase in the average C_T_ in spheroids compared to monolayers (C_T_=32.1 ± 0.52 and C_T_=30.4 ± 0.57, respectively), indicating a decreased level of miR-146a-5p in spheroids. According to our model, this should result in decreased EMMPRIN expression, as we have observed. However, contrary to our observations, reduced EMMPRIN should have led to decreased TGFβ levels, and reduced miR-146a-5p levels should have led to increased TRAF6 expression. Therefore, we suggest that the regulation of TGFβ and TRAF6 is more complex, and that miR-146a-5p or EMMPRIN are not their only regulators. For example, hypoxia was shown to enhance TGFβ expression (74), whereas TRAF6 was inhibited by hypoxia (75), explaining the absence of TRAF6 in all cells seeded as spheroids. Thus, it is possible that for some target proteins such as TGFβ and TRAF6, the effects of hypoxia outweigh the effects of EMMPRIN and miR-146a-5p, indicating that the net effect of all microenvironmental components should be considered.

In conclusion, we have demonstrated how spheroids enable us to better delineate the role of EMMPRIN on tumor progression. By knocking down EMMPRIN expression, we demonstrated that the many processes of metastatic tumor cells that EMMPRIN is involved in are more pronounced in spheroids. EMMPRIN reduced proliferation and angiogenesis, and increased drug resistance, invasion, EMT and dormancy. We also demonstrated that EMMPRIN contributes to the determination of cell morphology. The reduced expression of EMMPRIN in CT26-KD cells reveals that EMMPRIN is a gatekeeper that prevents entry of cells into dormancy, and helps maintain their hybrid EMT status, as we have shown before (36). Interestingly, we identified a negative feedback loop between miR-146a-5p and EMMPRIN, which may partly explain how miR-146a-5p is affecting EMT and metastasis. Thus, we recommend using the spheroids 3D platform for the study of the different mechanisms of tumor progression and especially of metastatic outbreak, and the continued study of EMMPRIN as a potential therapeutic target that can, both directly and via its effects on miR-146a-5p, push cells towards dormancy and prevent the metastatic outbreak.

## Data availability statement

The original contributions presented in the study are included in the article/**Supplementary Material**. Further inquiries can be directed to the corresponding author.

## Ethics statement

Ethical approval was not required for the studies on animals in accordance with the local legislation and institutional requirements because only commercially available established cell lines were used.

## Author contributions

GF: Investigation, Writing – original draft. ES: Investigation, Writing – original draft. PB: Writing – review & editing. MR: Conceptualization, Formal Analysis, Funding acquisition, Supervision, Writing – original draft, Writing – review & editing.
