# Supplementary material for: EMMPRIN promotes spheroid organization and metastatic formation: comparison between monolayers and spheroids of CT26 colon carcinoma cells
Source: Front Immunol. 2024 Apr 25;15:1374088. doi: 10.3389/fimmu.2024.1374088 (PMC11079191; doi:10.3389/fimmu.2024.1374088)
Supplement: Supplementary file 1 [file DataSheet_1.docx]

EMMPRIN promotes spheroid organization and metastatic formation: comparison between monolayers and spheroids of CT26 colon carcinoma cells

**Supplementary Data**

**Table S1: Four EMMPRIN target sequences used to knockdown its expression**

| **Location*** | **Gene** |
| --- | --- |
| 364-384 | 5'- CGACCTGCATACGAAGTACAT |
| 556-576 | 5'-CCCTCCTATTACAGATTGGTT |
| 611-631 | 5'- GCAATCACCAATAGCACTGAA |
| 829-849 | 5'- CCTGGTGTTGGTTACCATCAT |

***,** the locations are based on the accession number NP_001070652.1 of EMMPRIN isoform 2


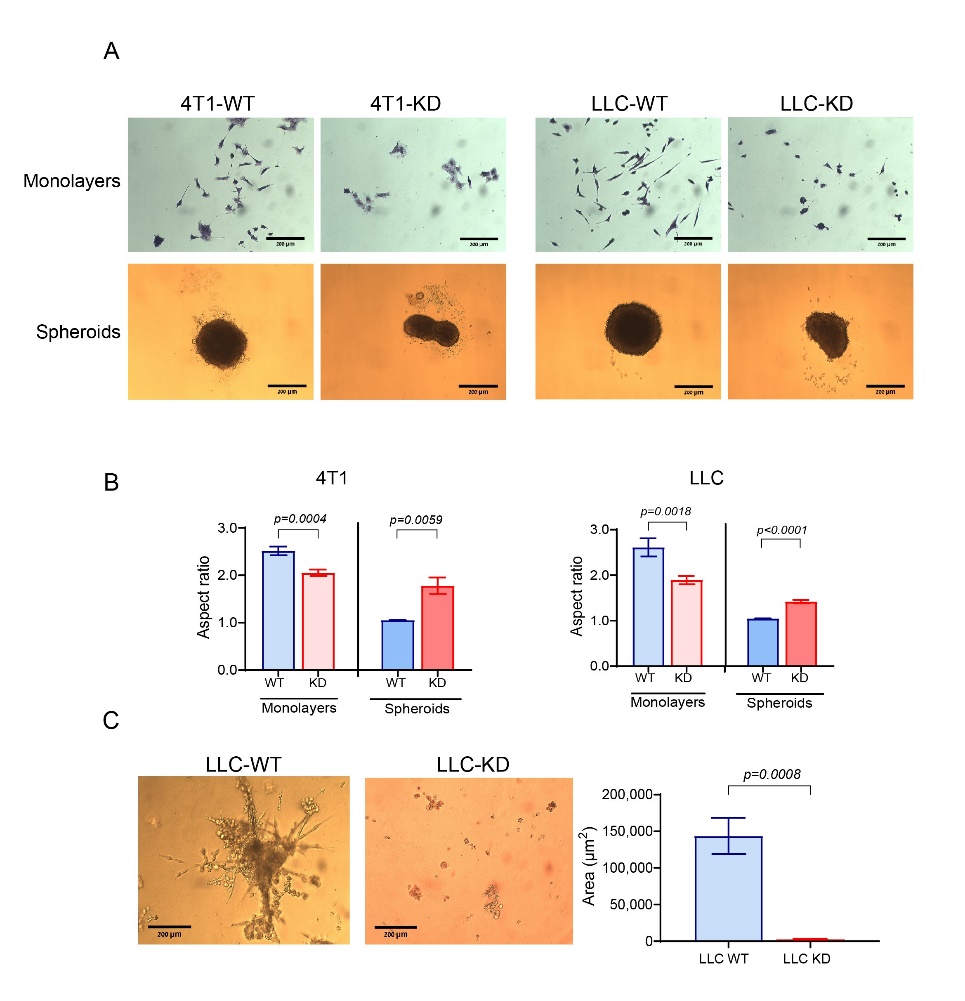


**Figure S1:** **Reduced EMMPRIN expression changes 4T1 and LLC cell morphology.** **(A)** The 4T1-WT and LLC-WT were transfected with the EMMPRIN-siRNA lentivirus vector, as described in the methods for CT26-WT cells. Representative images of 4T1-WT, 4T1-KD, LLC-WT and LLC-KD cells that were seeded (3x10^3^ cells/well/100μL) as monolayers. After 48 h of incubation in serum-starvation medium, cells were stained with 0.1% crystal violet, bar size 200μm. The EMMPRIN-KD cells are more spread and adhered to the culture dish compared with WT cells. Alternatively, cells were seeded as spheroids (5x10^3^ cells/well/100μL) on 1% agarose and allowed to grow for 3 days in full medium. Representative images of the spheroids are presented, bar size 200μm. EMMPRIN-KD cells form elongated aggregates, in contrast to the spheres generated by EMMPRIN-WT cells. **(B)** Analysis of the aspect ratio of cells seeded as monolayers or spheroids (n=18-24 for monolayers, n=4-7 for spheroids). Data are presented as means ± SEM, and analyzed by one-way ANOVA followed by Bonferroni's post-hoc test. **(C)** LLC-WT and LLC-KD cells were seeded on solid Coultrex®-coated 96-well plates (10^3^/well) in 60μl assay medium containing 2% BME and 2% FCS. Every 3-4 days the cells were re-fed with 30μl of the assay medium, and cells were incubated for a total of 13 days and then images were taken (bar size 200μm) and the area of the cell aggregates was measured (n=5). Data are presented as means ± SEM, and analyzed by one-way ANOVA followed by Bonferroni's post-hoc test.
